# Vascular mapping of the retroauricular skin – proposal for a posterior superior surgical incision for transcutaneous bone-conduction hearing implants

**DOI:** 10.1186/s40463-016-0181-z

**Published:** 2017-01-17

**Authors:** Adam Perenyi, Zsofia Bere, Janos Jarabin, Balazs Sztano, Edit Kukla, Ziad Bikhazi, Laszlo Tiszlavicz, Ferenc Toth, Jozsef Geza Kiss, Laszlo Rovo

**Affiliations:** 1 Department of Otorhinolaryngology, Head and Neck Surgery, University of Szeged, Tisza Lajos krt. 111, H-6725 Szeged, Hungary; 2 Affidea Diagnosztika kft., Semmelweis u. 6, H-6725 Szeged, Hungary; 3 Department of Pathology, University of Szeged, Állomás u. 2, H-6725 Szeged, Hungary

**Keywords:** Ear surgery, Conductive hearing loss, Numbness, Skin necrosis, Bone-anchored hearing aid, Arterial network, Temporal parietal soft tissue, Doppler ultrasound

## Abstract

**Background:**

Passive transcutaneous osseointegrated hearing implant systems have become increasingly popular more recently. The area over the implant is vulnerable due to vibration and pressure from the externally worn sound processor. Good perfusion and neural integrity has the potential to reduce complications. The authors’ objective was to determine the ideal surgical exposure to maintain perfusion and neural integrity and decrease surgical time as a result of reduced bleeding.

**Methods:**

The vascular anatomy of the temporal-parietal soft tissue was examined in a total of 50 subjects. Imaging diagnostics included magnetic resonance angiography in 12 and Doppler ultrasound in 25 healthy subjects to reveal the arterial network. Cadaver dissection of 13 subjects formed the control group. The prevalence of the arteries were statistically analyzed with sector analysis in the surgically relevant area.

**Results:**

The main arterial branches of this region could be well identified with each method. Statistical analysis showed that the arterial pattern was similar in all subjects. The prevalence of major arteries is low in the upper posterior area though large in proximity to the auricle region.

**Conclusions:**

Diverse methods indicate the advantages of a posterior superior incision because the major arteries and nerves are at less risk of damage and best preserved. Although injury to these structures is rare, when it occurs, the distal flow is compromised and the peri-implant area is left intact. Hand-held Doppler is efficient and cost-effective in finding the best position for incision, if necessary, in subjects with a history of surgical stress to the retroauricular skin.

**Trial registration:**

This was a non-interventional study.

## Background

Among all osseointegrated hearing implant systems, percutaneous abutment connection bone conduction systems have prevailed in the market for many decades. More recently, the newly developed transcutaneous magnet-connection bone conduction systems have gained increasing popularity. Implanted bone conduction devices that vibrate the bone via the skin are considered passive and may be referred to as “skin-drive” as opposed to “direct drive” systems [1].

Passive transcutaneous skin-drive systems with improved speech processors adequately treat mixed types of hearing loss associated with a relatively small sensorineural component. The surgery is straightforward, fast, and can be performed either in local or general anesthesia. An obvious advantage of the transcutaneous systems compared to the percutaneous ones is that the skin over the implant is left intact; thus, patients are more likely to accept the cosmetic results and adverse events can be prevented effectively (e.g. tissue overgrowth, peri-implant skin necrosis, numbness, or extrusion of the device [1]).

Potential drawbacks include the two magnets that put static and dynamic pressure on the skin that lies in-between, and the significantly larger surgical exposure in contrast to that used for the percutaneous implants. Specifically, compared to the punch technique [2] used for percutaneous implants, transcutaneous implants require a significantly larger skin incision.

Major injuries to the macrocirculation are strictly connected with compromised microcirculation, which results in compromised vitality of the skin flap, pain, numbness, and discomfort wearing the device [3]. Furthermore, blood flow in the skin is crucially controlled by perivascular nerves [4]. Consequently, injury of the neurovascular system leads to damaged perfusion of the given skin area. Skin erythema, discomfort, pain and hematoma have been reported [5].

An ideal incision leaves the normal vascular and neural system intact. Accurate knowledge of the vascular and neural system is therefore crucial.

Although the vascular anatomy of the temporal parietal soft tissues has been investigated [6–16], the studies are mainly concerned with reconstructive surgical aspects but not with the special aspects of hearing implant surgeries.

The null hypothesis formulated was that a large retroauricular incision injures the major arteries that feed the temporal parietal skin. The authors’ aim was to potentially decrease the surgical time and complication rate for magnet connection transcutaneous bone-conduction implants by (1) determining the preferable surgical incision, (2) finding the best suitable imaging method to determine the individual vascular and neural anatomy of the soft tissues of the retroauricular temporal-parietal region in a large number of subjects (50 in total) and (3) determining whether individual preoperative imaging is desirable.

## Methods

The protocol of the investigation was approved by the Institutional Review Board (Human Investigation Review Board, University of Szeged, Albert Szent-Györgyi Clinical Centre. Reference number: 3281), and the investigators obtained written informed consent from each participant or each participant’s guardian. All procedures performed in studies involving human participants were in accordance with the ethical standards of the institutional and/or national research committee and with the 1964 Helsinki declaration and its later amendments or comparable ethical standards.

Implanted subjects received the Cochlear ™ Baha ® 4 Attract System for this study, which is a transcutaneous bone-conduction hearing device reimbursed by our social security system.

Cadaver dissection was regarded as the gold standard of the investigations. Retrospective analysis of computed tomography angiography of the carotid arteries was found inefficient in visualizing some arteries, typically the posterior auricular artery. Angiography is a sensitive method that involves ionizing radiation. An alternative investigational method of the superficial vascular anatomy is in vivo photoacoustic imaging which provides high resolution 3D images of the vascular structure, the maximum depth is only 5 mm though [17].

Given the potential drawbacks of ionizing radiation, magnetic resonance angiography and ultrasound, both free of ionizing radiation, were selected for vascular studies.

The vascular pattern and blood flow of the temporal-parietal soft tissue was examined in 50 subjects. The examinations were performed with cadaver dissection (13 subjects), magnetic resonance angiography (12 subjects), and in vivo Doppler ultrasound (25 subjects). In order to rule out duplication of the results from individual symmetries, thus making the statistics more robust; only one side of each subject was examined. General exclusion criteria from the study were a history of a previous ear surgery and considerable stenosis of the carotid arteries.

### Cadaver dissection

The arteries of the temporal-parietal region were dissected in 13 adult cadavers. Authorized staff assigned them consecutively to this study as they became available. The study group comprised six men and seven women between the ages of 45 and 88 years, the mean age was 73.8 years. With the cadaver in the supine position and the head turned to the opposite side, a skin incision was made anterior to the tragus and continued anterior upwards to the skull and downwards under the tip of the mastoid process and along the anterior rim of the sternocleidomastoid muscle. Binocular surgical loupes were used for preparation. After visualization of the common carotid artery, the artery was followed distally to the carotid bifurcation and then to the external carotid artery. After the occipital, the posterior auricular and the superficial temporal arteries were detected at their origin, their distal sections were dissected as far as visually possible.

**Fig. 1:**
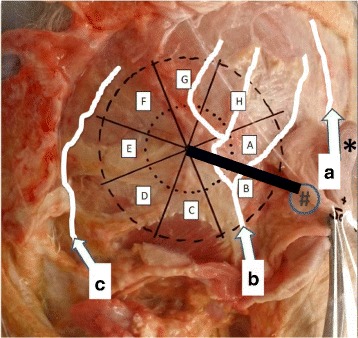
An example of the anatomical dissection shows the plastic template with the eight identical sectors and the three nutritive arterial branches of the external carotid artery (*white lines*): superficial temporal (*a*), posterior auricular (*b*), and occipital (*c*) arteries. The right auricle has been undermined and folded to the front. The EAC of the right ear is marked with “#” and the helix with “*”. The *arrows* show the direction of blood flow. The *thick black line* is a 6 cm long section between the external auditory meatus and the anticipated place of the screw and is the border of sectors *A* and *B*

Following the size data and incision recommendations of the manufacturer (Cochlear ™ Baha ® 4 Attract System Surgical Procedure, www.cochlear.com, accessed on 30 June 2015), a template of transparent film was made. This round template was 57 mm in diameter and its center (which indicates the implanted screw) was placed 5–6 cm from the external auditory meatus. The surface area of the template was subdivided into eight equally sized sectors, see Fig. 1. The line between the center and the external auditory meatus defined the border between sectors A and B. The transparent template with the eight sectors was again placed on the head. Photo documentation was made, which was followed by digital picture analysis.

### Retrospective analysis of magnetic resonance angiography (MRA) studies

A total of 12 consecutive MRA studies (1.5 Tesla, General Electric, USA) of the carotid arteries of adults who had undergone medical imaging due to possible arteriosclerosis of the internal carotid artery branches with findings of no stenosis, were analyzed retrospectively. The standard angiography protocol was: Axial T2 and FLAIR sequences, followed by a 3D TOF SPGR, then a sagittal and coronal T1 weighted image was added. The 3D TOF sequence’s scanning parameters included TE out of phase 6.8–13.1, TR 33 ms, Flip Angle 20, Bandwidth 25, FOV 24 cm. A slice thickness of 1.4 mm with an overlap of 15 mm, Acquisition timing: Freq 384, Phase 224, Phase FOV 0.75. The number of acquisitions was 1, Relative SNR 70%, maximum number of slices 256. The right occipital, posterior auricular and superficial temporal arteries and their branches were marked. The template, as described above in the cadaver examination section, was placed onto the digital images and analyses were performed by size of the arteries per sector.

### Doppler ultrasound

The parietal temporal region of 25 healthy adult subjects was examined with color Doppler ultrasound (20 subjects) and with preoperative portable handheld Doppler (5 subjects).

A total of 20 healthy adult volunteers who visited the clinic for diverse reasons and agreed to participate, were enrolled into the Doppler ultrasound study. The study group comprised 10 men and 10 women aged between 21 and 65 years with a mean age of 41.3 years. As the region of interest was not shaved, ultrasound gel was abundantly applied as a coupling agent between the probe and the skin. The right side was scanned. The 14 MHz linear array probe of a GE Logic 7 (General Electric Inc., USA) was used. The retroauricular temporal-parietal skin was scanned according to sectors A to H as shown in Fig. 1 by carefully scanning with the probe over each sector. Pressure to the skin that may cause squeezing of the vessels was avoided. In the B-mode (14 MHz, high magnification), the skin, the subcutaneous fat, the muscular layer and the periosteum were identified. The color mode with very sensitive parameters (overgained color, low-pulse repetition frequency) allowed for identification of the vessels. The largest artery in diameter was tracked. When a vessel was found, the probe was adjusted into its longitudinal axis to assess flow type and velocity and differentiate between arteries and veins. Later on, the probe was rotated by 90° to visualize the cross-section of the arteries. The largest artery diameters were noted in each preformed area; flow direction was determined and the diameter of the largest artery was noted.

#### Preoperative Doppler scans with a handheld Doppler device

A total of five consecutive subjects who were scheduled for Baha Attract implantation were enrolled into this preoperative assessment. A portable handheld Doppler device (Hadeco Doppler Smartdop 50ex) was used to identify and mark the main arterial trunks just prior to surgery to help the surgeon determine the best position of the incision and the implantable hearing aid. The results were investigated case-by-case.

Statistical analysis was done with the above described template in the cadaver (1), MRA (2), and Doppler ultrasound (3a) evaluations, whereas a case-by-case analysis was performed for the handheld Doppler assessments (3b).

Statistical analysis was performed with SigmaPlot 13 scientific graphing and statistical analysis software (Systat Software Inc, California). Data are presented in mean ± SE. For inter-group comparisons, Kruskal-Wallis One Way Analysis of Variance on Ranks was performed (*H* = 52,047 with 7 degrees of freedom; *p* <0.001) with Dunn’s multiple comparison method (*p* <0.05).

## Results

Based on our individual results with all methods, our findings agree with previous observations of the main arterial blood supply to the temporal parietal skin reported by several authors [6–16]. Cadaver dissection, MRA and Doppler ultrasound proved to be good methods to locate the most important external carotid artery branches; i.e. posterior auricular artery, superficial temporal artery, and occipital artery. In several cases, another considerably large branch was found that springs off the superficial temporal artery or the parietal branch of the superficial temporal artery and forms a vascular anastomosis together with the posterior auricular artery. This is consistent with the observations of Kobayashi et al. [9].

The results demonstrate the location and arterial pattern in the temporal parietal region (Fig. 1). It is clearly visible that the relatively large arteries, i.e. the posterior auricular artery and a postauricular branch of the superficial temporal artery, are likely to be found in the strip close behind the auricle. Via the occipital access, the occipital artery was found. In contrast, the area between the above arteries, i.e. the posterior superior parietal region, is relatively poor in arteries. The blood flow is directed from the center (external carotid artery) to the periphery, as shown with the arrows (Fig. 1).

**Fig. 2:**
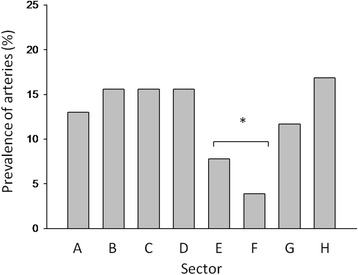
Prevalence of arteries that were detected with cadaver dissection in each sector, A to H for 13 cadaver specimens

**Fig. 3:**
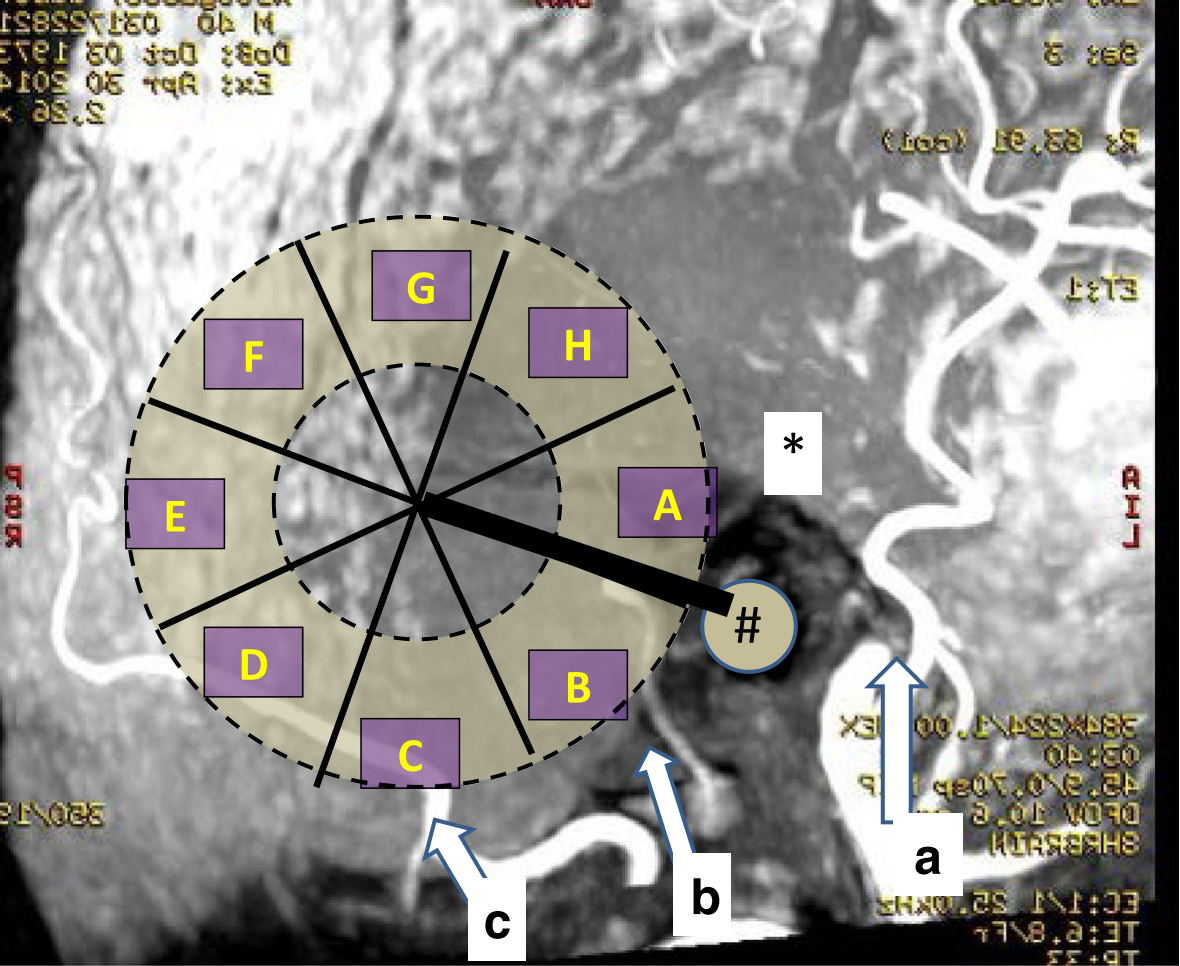
An example of the MRA study. The plastic template with the eight identical sectors and the three nutritive arterial branches of the external carotid artery (*white lines*) are shown: superficial temporal (*a*), posterior auricular (*b*), and occipital (*c*) arteries. The EAC of the right ear is marked with “#” and the helix with “*”. The *arrows* show the direction of blood flow. The *thick black line* is a 6 cm long section between the external auditory meatus and the anticipated place of the screw and is the border of sectors *A* and *B*

**Fig. 4:**
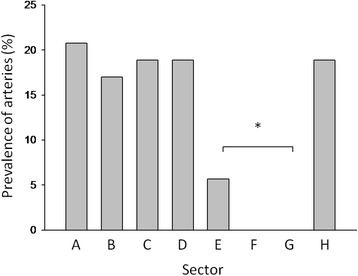
Prevalence of arteries that were detected with magnetic resonance angiography in each sector, *A* to *H* for 12 living subjects. Sectors *E*, *F*, and *G* are poorly vascularized

The prevalence of arteries that were detected with cadaver dissection in each sector (see Fig. 2) correlate well with those obtained via MRA of the twelve living subjects (see Figs. 3 and 4).

All major arteries were successfully detected with the retrospective MRA studies of the carotid artery. An advantage over computed tomography angiography is that bone has no signal on MRA, thus the arteries can be visualized better and no exposure to ionizing radiation occurs. The results were consistent with those of our cadaver study; i.e. the relatively large arteries are likely to be found in sectors A, B, C, D, and H, whereas sectors E, F, and G are relatively poorly vascularized (Figs. 3 and 4).

**Fig. 5:**
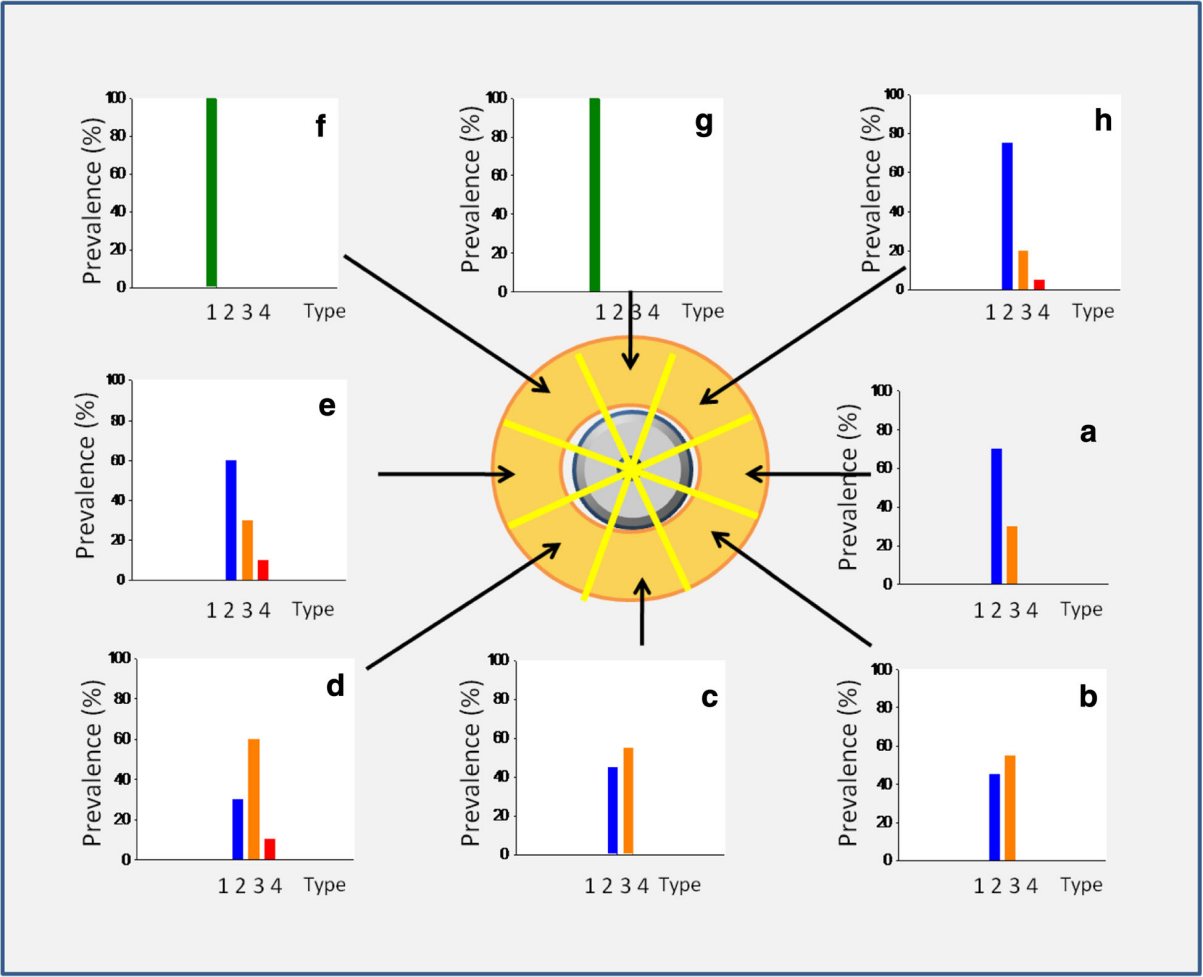
Prevalence of the largest artery in each sector, measured by Ultrasound for 20 volunteers. The chart gives a detailed percentage overview of intraluminal arterial diameters in each sub-region. Artery types: type 1: 0–0.5 mm, type 2: 0.5–1 mm, type 3: 1–2 mm, type 4: >2 mm

The Doppler study could precisely confirm the results of the cadaver and MRA studies. Arteries of the largest diameter were found in sectors A, B, C, D, E, and H, which are comparable with the location of the main arterial trunks, i.e. posterior auricular, superficial temporal and occipital arteries (Fig. 5).

The differences in the results from MRA and Doppler ultrasound may arise from the individual variance and examination technique. A 1.5 Tesla MRA seems to be less sensitive than the cadaver dissection.

During the portable handheld Doppler evaluation, an artery was detected in sector G in one out of five subjects and none in sector F.

**Fig. 6:**
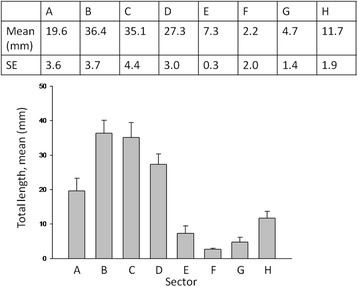
Total length of visible arteries in each sector (mean ± SE [mm]) on cadaver dissection (*n* = 13) and magnetic resonance angiography (*n* = 12). The total length of the arteries in the posterior superior sectors (*E*, *F*, and *G*) is significantly lower than in the other sectors. (Dunn’s method, *P* ≤ 0.001)

The sum of arterial lengths proved to be highest in sectors B and C (Fig. 6) because the posterior auricular and the occipital arteries enter the retroauricular temporal region there. Also these sectors contain the large arteries and, therefore, they have to be considered as they are situated in the high surgical “danger zone”. The upper and posterior sectors (E, F, and G) are less dangerous because of the low sum-total length and small diameter of the arteries. The analysis of the total lengths of prevalence of arteries in the upper posterior sectors (E, F, and G) is significantly lower than in the retroauricular and inferior sectors (B, C, and D). Sectors A and H are transient zones, because the difference to the other sectors was not significant, but the tendency is that the prevalence of arteries compared to the retroauricular and inferior sectors (B, C, and D) is higher.

## Discussion

Although the vascular anatomy of the temporal skin and the temporal muscle has already been studied, these investigations focused on the plastic surgical aspects only [6–16]. To our knowledge, our study is the first in the literature with the aim to reveal the vascular pattern of the retroauricular temporal parietal area to optimize the position of the surgical incision.

Our study suggests that, apart from individual variations, the average tendency is that the larger arterial branches are close to the auricle, whereas the superior posterior area of the temporal parietal region contains small arteries only.

An optimal incision preserves good vascular capacities, i.e. the proximal (outflow) sections. Less bleeding requires less coagulation and, thus, the surgical time can be decreased. Ideally, the incision is short and placed where the arteries and nerves have split already into their smaller branches. The official suggestion by the manufacturer Cochlear® is a 7.5 cm curved incision behind the auricle and 15 mm apart from the rim of the internal magnet plate (Cochlear ™ Baha ® 4 Attract System Surgical Procedure, www.cochlear.com, accessed on 30 June 2015). A 7.5 cm long incision crosses three sectors with two major arteries. Our results suggest that this causes considerable injuries on both macrovascular and, consequently, microvascular levels. If a similar incision is made in the posterior superior area, the risk of traversing a major artery is limited.

The manufacturer’s recommendation should be revised as it puts the skin laying between the internal and external magnets at considerable risk because both of the main nourishing arteries (posterior auricular artery and posterior branch of the superficial temporal artery) in their proximal section are prone to injury by the 7.5 cm incision. If the upper posterior arteries are transected, it is likely that only one, i.e. either the occipital or the posterior auricular artery branches, are transected but, importantly, in their distal outflow sections, after the main outflow tract has formed several branches and anastomosis is already apparent. This means that the surgical area over the implant retains its intact perfusion.

Although the neural network of the skin was not investigated in our study, the anatomical atlases [e.g. Sobotta: Atlas of Human Anatomy, Urban&Fischer, 14th edition] suggest that the major nerves run along the same pathway with the major vessels, thus they are prone to injuries with the same likelihood. Knowing this, it is advantageous to place the incision further towards the upper posterior area so that injuries to the C-fibers (non-myelinated fibers) and the sensory fibers of the skin occur in the distal section, thus only the distal small area will be affected by microcirculatory and sensory compromises.

**Fig. 7:**
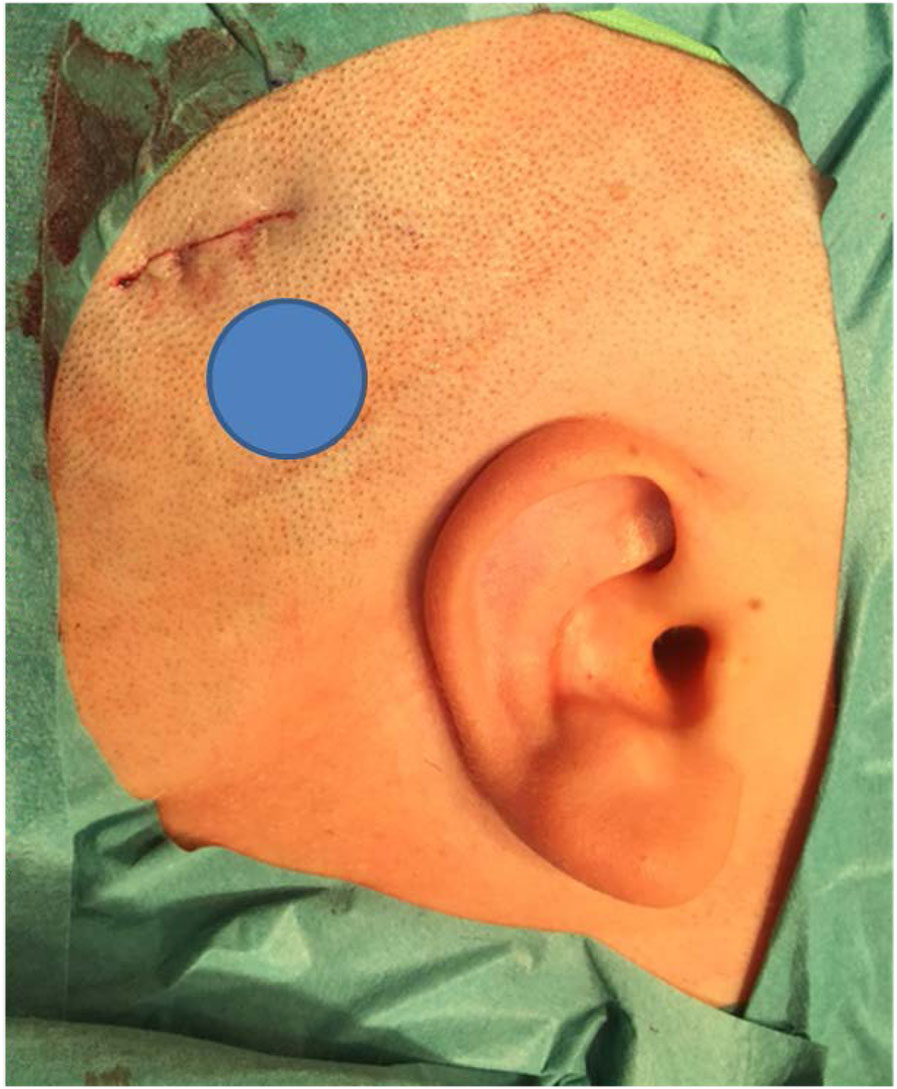
The retroauricular skin and soft tissues are elastic and can be retracted enough and there is enough space to complete each step of the implantation from a 2.5–4 cm slightly curved incision in the posterior superior temporal-parietal region

Our results clearly reveal that it is not possible to conduct such surgeries without transecting at least one major artery with the given 7.5 mm incision, which underlines the necessity to make the incision as short as possible. In our experience, proper exposure can be achieved from a 3 to 4 cm curved incision in the upper posterior area by undermining and mobilizing the skin and soft tissues from the periosteum, not only over the implant but also distal to the incision line. The skin can be retracted enough, and there is enough space, to complete each step of the implantation, as shown in Fig. 7. Because there are no perforating vessels between the periosteum and the soft tissues, the blood supply of the flap will not be affected.

Our cadaver dissection and imaging studies confirmed that the vascular anatomy is stable in most of the cases, i.e. the posterior superior area is less vascularized than the anterior area. This makes not only the expensive (MRA) but also the easy-to-access and cost-effective preoperative imaging studies (Doppler) unnecessary and thus improves time efficiency. Those patients in which the vascular architecture has already been stressed from previous ear surgery (e.g. mastoidectomy, radical middle ear surgery) are challenging because the previous exposures have deteriorated the blood supply and further incisions diminish it even more [3]. In our opinion, preoperative Doppler examination performed with a simple portable handheld instrument is sufficient even after previous ear surgeries.

## Conclusion

Our study clearly confirms the coherent advantages of the short posterior superior incision when transcutaneous bone-conduction hearing devices are implanted, based on the surgical anatomical vascular relationships. Although this study involved passive magnet connection osseointegrated systems only, our recommendation for the ideal incision can be beneficial to avoid postoperative complications for the implant of active abutment connection osseointegrated systems also. The undeniable similarities of the anatomical vascular relationships among the individual subjects puts the necessity of preoperative imaging (not only the expensive MRA but also the cost-effective ultrasound) into question.

Our opinion is that preoperative imaging is recommended in those cases, only in which the retroauricular area was subject to previous surgical stress. In those cases, portable handheld Doppler examination is reliable, quick, and cost effective. In any case, the surgeon should aspire to make the shortest possible incision.
